# Liquid water intake of the lone star tick, *Amblyomma americanum:* Implications for tick survival and management

**DOI:** 10.1038/s41598-020-63004-9

**Published:** 2020-04-07

**Authors:** L. Paulina Maldonado-Ruiz, Yoonseong Park, Ludek Zurek

**Affiliations:** 1 Department of Entomology, Kansas State University, 123 West Waters Hall, Manhattan, KS 66506 USA; 2 Central European Institute of Technology, Center for Zoonoses, University of Veterinary and Pharmaceutical Sciences, Palackeho 1946/1, Brno, 612 42 Czech Republic; 3 Department of Chemistry and Biochemistry, Mendel University, Zemedelska 1, Brno, 613 00 Czech Republic

**Keywords:** Zoology, Animal behaviour

## Abstract

Ixodid ticks are ectoparasites that feed exclusively on blood as their source of nutrients. Although ticks spend most of their life off the host, until now it has been assumed that the blood and the water vapor are the only sources of water to maintain water balance and prevent desiccation. Here we report for the first time that adult lone star ticks, *Amblyomma americanum*, also actively drink nutrient-free water, which greatly increases their survival. The volume of ingested water is greater in females than males (0.55 ± 0.06 vs 0.44 ± 0.07 µl) and most likely due to differences in tick size. Water uptake occurs through mouthparts and it can be later observed in the salivary glands and the midgut. We also exploited this behavior by adding a variety of inorganic compounds and microorganisms to water. Addition of inorganic salts to drinking water such as KH_2_PO_4_ + NaCl+KNO_3_ resulted in 100% tick mortality within 3 days. As a proof of concept for using the water drinking as a delivery route of toxic reagents for ticks, we also show that adding *Pseudomonas aeruginosa* to drinking water quickly leads to tick death. This tick behavior can be exploited to target important physiological systems, which would make ticks vulnerable to dehydration and microbial dysbiosis.

## Introduction

Ticks are obligate blood feeding ectoparasites and one of the most important arthropod groups that vector pathogens to people and other animals worldwide^1–5^. Hard ticks have to survive extended periods off the host (~ 90% of their lifetime) throughout their development. Therefore, tick survival is dependent on their ability to maintain water balance, as they have to overcome fluctuating environmental conditions, primarily temperature and relative humidity (RH)^6,7^. Ticks maintain water balance by water vapor uptake. Water vapor uptake directly depends on the Critical Equilibrium Activity (CEA), which is the minimum RH required for water uptake and maintaining the water balance in the body^8–10^. In most ixodid ticks the CEA is close to 90% RH^10^; in *Amblyomma americanum* water vapor uptake is between 85% and 93% RH and accounts for up to 77% of the water taken in^11^. Water vapor uptake was first reported in *Amblyomma variegatum* with the mouthparts as the main site of this uptake^12^. It was demonstrated that the salivary glands secrete to the mouthparts a hygroscopic solution high in chlorine, potassium, and sodium^6,13^ which absorbs water vapor that is then ingested by the tick^6^.

Off host ticks could also actively drink water to maintain water balance, whereas several studies reported no water drinking behavior in *Ixodes ricinus, Dermacentor marginatus*, and *D. reticulatus*^14–16^. Our recent study demonstrated that *I. scapularis* can approach and touch water droplets and occasionally insert the hypostome and drink water (5% of ticks tested)^17^. Drinking-like behavior was also observed for several *Rhipicephalus* species*, Dermacentor nuttalli, Amblyomma hebraeum*, and larvae of *A. americanum*^18–20^. However, the significance of liquid water drinking in tick biology and fitness and its potential for management have not been investigated.

In this study, we aimed to determine 1) whether adults *A. americanum* ingest water; 2) the volume of water taken in and its distribution within the tick; 3) the significance of water drinking to tick survival; and 4) the potential for exploitation of water drinking behavior for tick management.

## Materials and Methods

### Ticks

Non-fed, 2–3 months old adults of *A. americanum* were obtained from the Oklahoma State University tick rearing facility and kept at 4 °C and >95% RH to minimize tick activity until used for experiments. Before each experiment, ticks were preconditioned for 24 hrs at 28 ± 3 °C and 30 ± 5% RH in individual 50 × 9 mm Tight-Fit Lid Dishes (Falcon Brand Products, New York, USA).

### Water drinking behavior

#### Laboratory-reared *A. americanum*

After preconditioning, ticks (20 males, 20 females) were placed individually into sterile 50 × 9 mm Tight-Fit Petri dishes and provided with a 5.0 µl droplet of deionized sterile water and kept at 40% RH and 26 °C (laboratory conditions during experiments). Ticks were observed for 1 hour. Ticks that stayed in contact with droplet after 1 hour were left in the same dish until the next day. Ticks that did not drink during the first hour were transferred to a new dish without water, kept at 28 °C ± 3 °C, and provided with a 5.0 µl water droplet every 24 hrs until they were observed to drink water.

#### Field collected ticks

Questing adult ticks were collected from northeastern Kansas (Konza Prairie Biological Research Station) and southeastern Kansas (around Pittsburg) by cloth dragging. A total of 88 adult ticks were collected; 56 *A. americanum*, 30 *Dermacentor variabilis*, and 2 *A. maculatum*. Upon collection, ticks were kept in a cooler with ice (3–5 °C) and over 95% RH until arrival to the laboratory. Water drinking behavior was assessed as described above for laboratory-reared ticks.

### Ticks survival with and without access to liquid water

Preconditioned laboratory-reared *A. americanum* adults were divided into 3 equal groups and placed individually in cardboard containers (volume: 236 ml, 8.5 × 4.8 cm) (Rigid Paper Corp.) modified with a sterile petri dish base on the bottom (100 × 15 mm Fisherbrand) and a screen nylon mesh on the lid of the cup. Three different treatments were used after preconditioning: Group 1 (access to water) was provided with a 5.0 µl droplet of deionized sterile water daily. Group 2 (without access to water) was also provided daily with a 5.0 µl water droplet to maintain the same level of humidity as in the Group 1, but the droplet was not accessible to the ticks; water droplet was protected by a metal nut (1 × 2 cm) covered by a screen nylon mesh. Group 3 (no water) was provided with no water. Bioassays were conducted separately for males and females (n = 10 for each) and 3 replicates for each group were performed for all treatments. All experiments were conducted at 28 ± 3 °C and 30 ± 5% RH (in same experimental area). Room conditions were set to 30% RH and 28 °C. During the experiments, conditions were recorded every 12 hours using a digital thermos-hygrometer (part 37950–03) (Cole-Parmer, IL, USA). Ticks were kept in same conditions (28 ± 3 °C and 30 ± 5% RH) between treatments and mortality was recorded every 24 hrs for 30 days (until 100% mortality in Groups 2 and 3).

### Quantification of water intake

In order to quantify the amount of drinking, two different approaches were used: a) bacteria mixed in the water to trace the amount of bacteria (and water) ingested, and b) capillary feeding showing the visually quantifiable volume of water. Ticks were preconditioned as described above.

#### Droplet drinking with bacteria

We used bacteria as a quantifiable tracer for the estimation of the liquid ingested amount. Deionized sterile water was inoculated with Gram-negative (*Escherichia coli*) or Gram-positive (*Staphylococcus epidermidis*) bacteria. Initial estimation of inoculum size was standardized by optical density at 600 nm using a BioMate 3 series spectrophotometer (Thermo Fisher Scientific, Waltham, MA) and confirmed by culturing on tryptic soy agar at 37 °C for 24 hours. Optic density at 600 nm was only used to measure turbidity (cell density) as a reference point to reach an approximation of 1,000 to 9,000 cells/µl. A 5.0 µl droplet of bacteria-inoculated water was provided to each preconditioned tick. The mean concentration of inoculum offered to each tick was 7.6 ± 0.7 × 10^3^ CFU (colony forming units)/µl (*E. coli*) and 4.8 ± 0.7 × 10^3^ CFU/µl (*S. epidermidis*). Ticks that drank from the inoculum droplet for 1 hour were processed (18 males and 20 females from each bacterial group). In order to minimize the loss of bacteria during the process, we harvested the bacteria immediately after the time allowed for the 1 hr drinking. Ticks were surface sterilized with 0.5% sodium hypochlorite, 70% ethanol, and washed with sterile water. Individual ticks were then cut in small pieces and homogenized in phosphate buffer saline. The homogenate was spread plated on tryptic soy agar and incubated at 37 °C for 24 hrs. We ensured that there were no culturable bacteria in the laboratory reared ticks by plating processed ticks on tryptic soy agar in an initial pilot test with 40 ticks. The volume of water ingested by ticks was determined by using the number of CFU obtained from the ticks and the bacterial inoculum size per µl. *Escherichia coli* and *S. epidermidis* were confirmed by colony and cell morphology and a rapid Gram staining test.

#### Capillary drinking

Preconditioned ticks (20 males and 20 females) were immobilized on dental wax and placed inside a Tight-fit lid 50 × 9 mm petri dish (5 ticks per dish). The tick hypostome was inserted into a polyimide capillary tube (454 µm diameter x 2 cm length). Deionized sterile water was pipetted into capillary tubes and dishes were sealed during the assays. The decrease in water volume was measured (1 mm = 0.1464 µl) every 15 minutes for 1 hr. Final measurements were recorded and an evaporation control (capillary tube without a tick) was subtracted from measurements to estimate the amount of water intake by ticks.

### Internal distribution of ingested water (droplet and capillary feeding)

Ticks were preconditioned before experiments as described above. One mM rhodamine 123 (Rh123, Sigma-Aldrich, St. Louis, MO, USA) was used as a fluorescent dye to determine the distribution of water within tick internal organs after droplet and capillary feeding.

**Figure 1:**
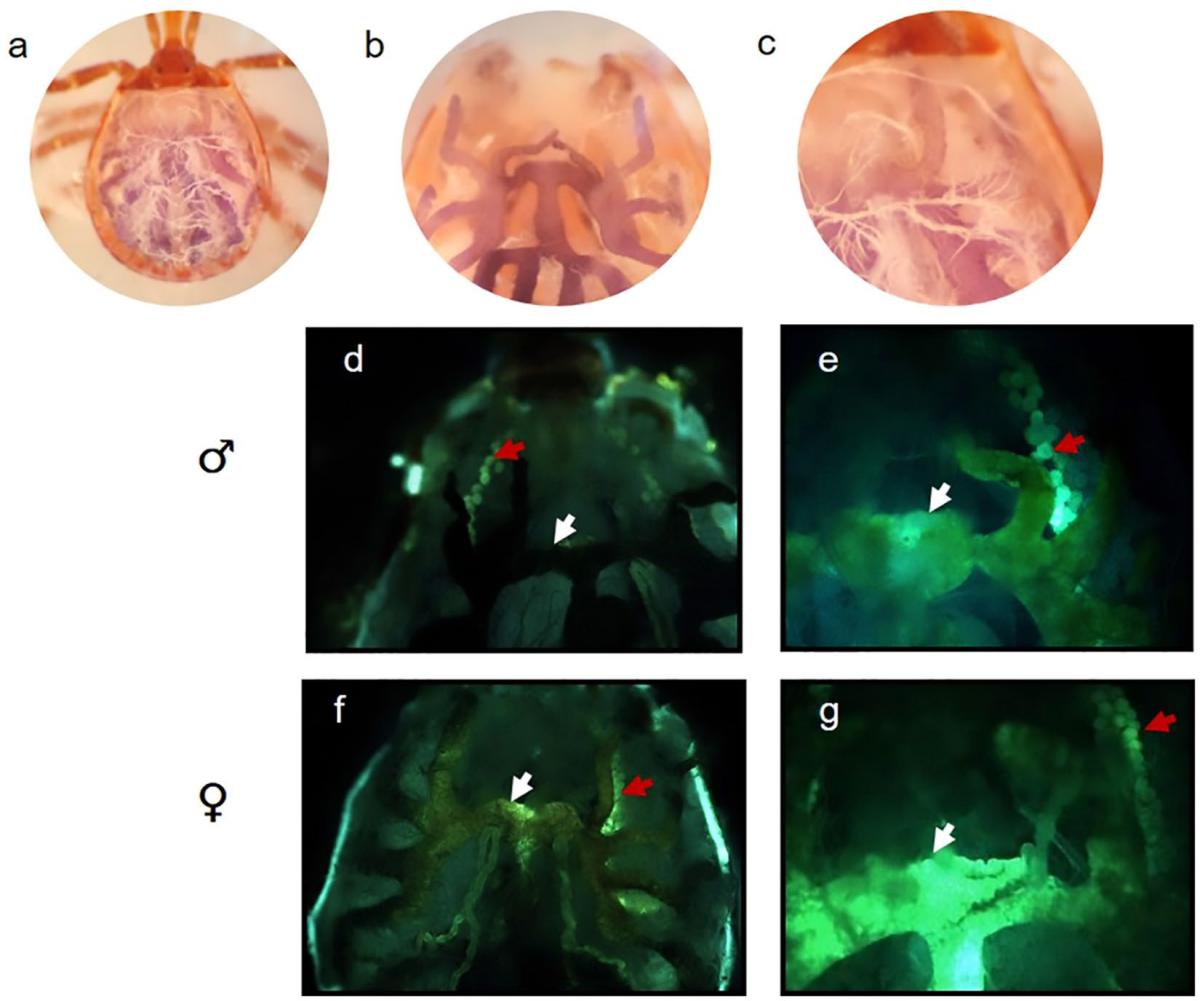
Water absorption organs in lone star ticks. From (**a–c**): images of internal morphology of the adult ticks representing the location of the fluorescence below. (**d**) Male after droplet feeding with Rh123 and (**e**) after capillary feeding with Rh123. (**f**) Female after droplet feeding with Rh123 and (**g**) after capillary feeding with Rh123. White and red arrows point to fluorescence in the midgut and the salivary glands, respectively.

Droplet and capillary feeding were conducted as described above with a few modifications. To minimize the loss of fluorescence, dishes with ticks were kept under dim light during bioassays and drinking time was reduced to 30 min (capillary feeding) and to 15 min (droplet feeding). After the exposure, ticks (4 males and 4 females for capillary feeding and 2 males and 3 females from droplet feeding) were washed with sterile water and immobilized on dental wax for dissection in phosphate buffer saline. The dorsal integument of ticks was removed with a surgical scalpel and images of internal organs (Fig. 1a–c) were captured using a camera (DFC400) attached to a stereo microscope (M205FA; Leica, Heerbrugg, Switzerland) with the GFP filter set (excitation BP470/40, dichromatic mirror 500, and emission filter 480LP) to visualize water by observing fluorescence in the internal tissues.

### Exploitation of water drinking behavior for tick management

Non-fed males and females of *A. americanum* were obtained from the Oklahoma State University Tick rearing facility and kept at 4 °C and 95% R.H. For all assays, ticks were preconditioned as described above.

#### Inorganic compounds

Different combinations and concentrations of inorganic compounds were tested to determine the optimal treatment to effect tick mortality (Tables 1 and S1). Based on the function of the salivary glands, we focused primarily on salts with potassium, sodium, and chlorine. Ticks were individually provided with a 5.0 µl droplet of a tested compound in sterile deionized water every 24 or 48 hrs. Control ticks were kept at the same conditions and were provided daily with a 5.0 µl droplet of deionized sterile water. Bioassays were conducted from 7 to 19 days and tick mortality was monitored daily.

**Table 1:** Summary of inorganic compounds tested and their impact on *Amblyomma americanum* survival after water droplet ingestion.

			Males		Females		Combined	
Treatment	Final concentration mM (compound ratio)	Length of bioassay (days)	*n*	% survival (days)	*n*	% survival (days)	*n*	% survival (days)
*Fert.20.10.20	2.6 (KH_2_PO_4_)/4.7 (KNO_3_)	7	5	100 (7)	5	100	10	100
*Fert.20.10.20 + NaCl	2.6/4.7/5	7	5	100 (7)	5	100	10	100
*KH_2_PO_4_ + NaCl	2/250	12	10	70 (12)	10	30	20	50
KH_2_PO_4_ monobasic	500	17	10	0 (6)	10	0 (17)	20	0 (17)
Na_2_HPO_4_ dibasic	500	17	10	0 (7)	10	0 (9)	20	0 (9)
NaCl	500	19	10	0 (8)	10	0 (9)	20	0 (9)
KH_2_PO_4_ monobasic	100	10	10	40 (10)	10	30	20	35
Na_2_HPO_4_ dibasic	100	10	10	25(10)	10	25	20	25
KH_2_PO_4_ + NaCl	1000 (1:1)	7	10	0 (6)	10	0 (4)	20	0 (6)
KH_2_PO_4_ + NaCl	125/62.5	7	5	0 (3)	5	0 (4)	10	0 (4)
(NH_4_)_2_PO_4_ + NaCl	50/5	7	10	0 (6)	10	0 (7)	20	0 (7)
KH_2_PO_4_ + NaCl + KNO_3_	120/20/20	7	10	0 (2)	10	0 (3)	20	0 (3)
KH_2_PO_4_ + NaCl + KNO_3_	60/10/10	7	10	0 (2)	10	0 (3)	20	0 (3)
KH_2_PO_4_ + NaCl + KNO_3_	50/5/5	7	10	0 (5)	10	0 (6)	20	0 (6)
^ɬ^KH_2_PO_4_ + NaCl + KNO_3_	240/40/40	7	8	0 (3)	8	0 (4)	16	0 (4)
^ɬ^KH_2_PO_4_ + NaCl + KNO_3_	120/20/20	7	8	0 (2)	8	0 (3)	16	0 (3)
^ɬ^KH_2_PO_4_ + NaCl + KNO_3_	60/5/5	7	10	0 (7)	10	0 (5)	20	0 (7)
^ɬ^KH_2_PO_4_ + NaCl + KNO_3_	50/5/5	7	10	0 (5)	10	0 (6)	20	0 (6)

^*^Recommended concentration from Peter’s fertilizer 20.10.20.

^ɬ^Every other day treatments.

#### Microorganisms

To assess tick survival after microbial ingestion, different bacterial species and strains in various dosages (Tables 2 and S2) were provided to ticks in a 5.0 µl water droplet. Control ticks were kept in the same conditions and were provided daily with a 5.0 µl droplet of deionized sterile water. Tick mortality was monitored every 24 hrs. For assays with *Pseudomonas aeruginosa*, exposure to the inoculum was limited to 1 hr and a one-time application. For other assays, the exposure time varied depending on the treatment (Table 2). After the microbial exposure, ticks were transferred to new dishes and given a 5.0 µl droplet of deionized sterile water on daily basis. Dead ticks from different treatments and a subset of control live ticks were processed to determine the presence of the microorganisms in the body. Ticks were surface sterilized using 0.5% sodium hypochlorite, 70% ethanol, and washed with sterile water. Individual ticks were cut in small pieces by sterile scissors and homogenized in phosphate buffer saline. Homogenates were spread plated on tryptic soy agar and incubated at 26 °C for 72 hrs.

**Table 2:** Summary of microorganisms tested and their impact on *Amblyomma americanum* survival after water droplet ingestion.

Microorganisms					
Treatment	Concentration (CFU/µl)	Days of exposure	Length of bioassay	*n* (♂,♀)	% survival
*Pseudomonas aeruginosa*	1.1 × 10^4^	1	7	15 (0,15)	0
	1.8 × 10^3^	1	7	17 (7,10)	53
	1.8 × 10^2^	1	7	16 (9,7)	81
***Bacillus thuringiensis***					
*ser* kurstaki	~1.5 × 10^4^	3(♂), 7(♀)	15	18 (8,10)	38.8
*ser* israelensis	1.5 × 10^4^	3(♂), 7(♀)	15	18 (8,10)	55.5
*ser* morrisoni	1.0 × 10^4^	3(♂), 7(♀)	15	18 (8,10)	61.1
*ser kurstaki	1.8 × 10^4^	7	7	10 (0,10)	70

^*^Bioassay with nymphs.

#### LD_50_ for P. aeruginosa

To determine LD_50_ in ticks, 3 different bacterial concentrations were used: 10^4^ CFU/µl, 10^3^ CFU/µl, and 10^2^ CFU/µl. Ticks were divided into 3 groups of 15 and preconditioned as described above. Ticks were immobilized on dental wax inside a Tight-fit lid 50 × 9 mm petri dish (5 ticks per dish and 1 water evaporation control). A polyimide capillary tube (454 µm diameter x 2 cm length) was inserted over the tick hypostome and filled with a bacterial suspension in sterile water (n = 10 for each treatment) or sterile water (n = 5) as control. The decrease in water volume was measured (1 mm = 0.1619 µl) every 15 minutes for 1 hr. Final measurements were recorded and evaporation control (a tube with no tick) was subtracted from this measurement to determine the amount of liquid intake. After 1.0 hr exposure, ticks were removed from the wax, placed individually in sterile petri dishes, and given a 5.0 µl droplet of deionized sterile water immediately after treatment and then every 24 hrs during the assay. Ticks were kept in 28 ± 3 °C at 30 ± 5% RH and mortality was recorded daily. All dead ticks were processed to determine the presence of *P. aeruginosa* as described above.

### Statistical analysis

Kaplan-Meier survival curves^21^ were generated using the data from surviving and dead individuals over time to assess median survival times of each treatment. Statistical significance between survival curves of treatments was determined using multiple statistical comparison approaches. Using p = 0.05 as cutoff for significance, we conducted Mantel-Cox, Genhan-Breslow-Wilcoxon and Chi Square tests using GraphPad Prism version 7.04 for Windows (GraphPad Software, California USA, www.graphpad.com).

## Results

### Water drinking behavior

All laboratory-reared ticks (n = 40) preconditioned at 30 ± 5% RH and 28 ± 3 °C for 24 hrs displayed water drinking behavior when a drop of water was offered over the course of 3 days (5 µl daily). Few ticks were not attracted to drink water on day 1; however, eventually drank on day 2 or 3. Two types of behavior were observed: 1) slowly approaching the water (5 to 20 min) and placing the hypostome into the water droplet or 2) immediately reaching the water droplet and in less than a minute inserting the hypostome into the water. In both cases, slow movements of chelicera and pulsatile movements on the base of the hypostome were observed, indicating water drinking. Ticks often exhibited squatting behavior on the water droplet and had the entire ventral surface of the body submerged in the water.

**Figure 2:**
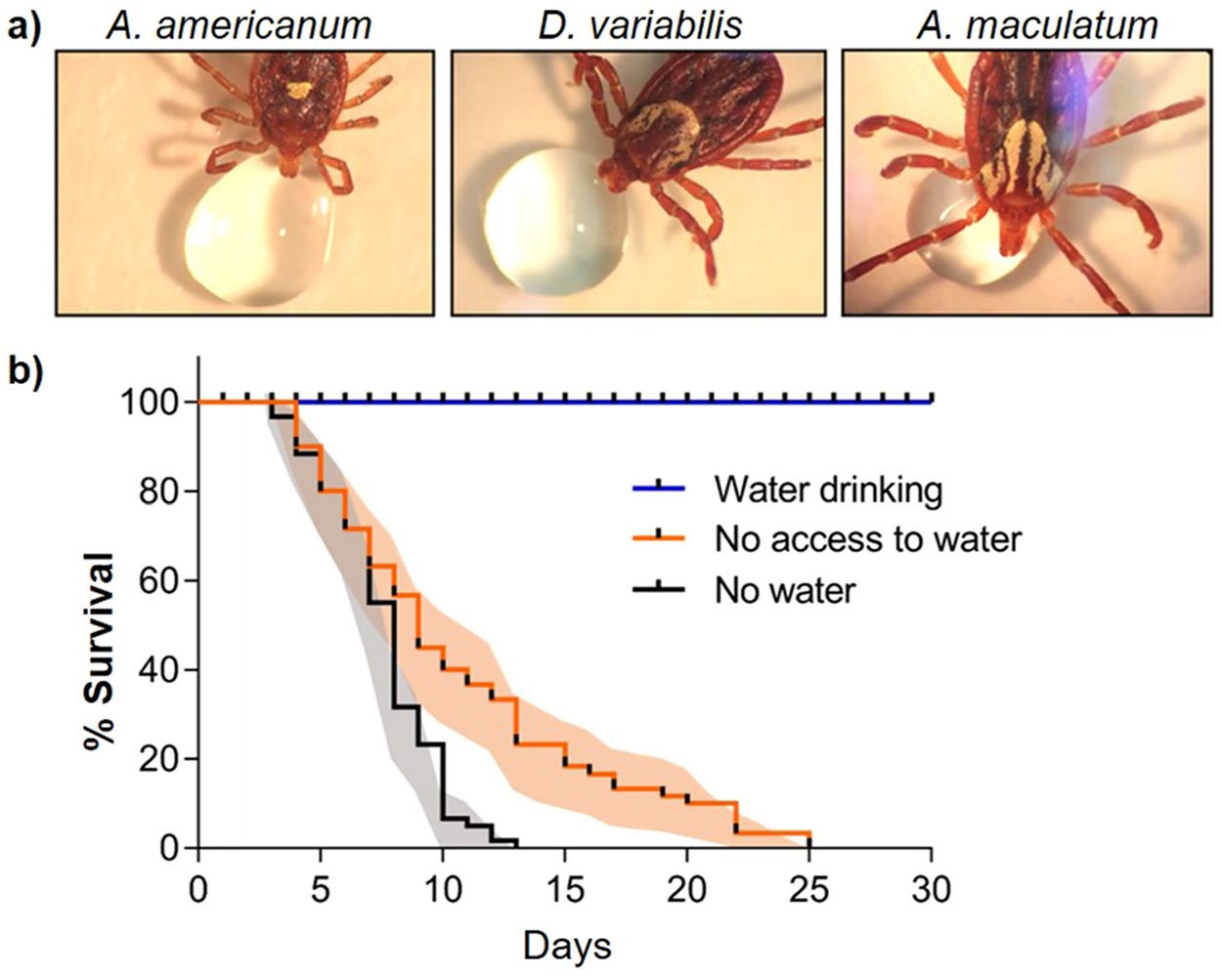
Water drinking and its impact in tick survival. (**a**) Water drinking behavior exhibited by adult field collected female ticks; (**b**) Impact of water drinking on male and female *Amblyomma americanum* survival (n = 60/treatment). Water drinking (direct contact), No access to water (no contact, water covered by screen mesh), No water: no droplet provided. Percent survival shown in Kaplan-Meier survival curves. Color shaded areas represent SEM for each data point.

The same behavior was observed in ticks collected in the field (Fig. 2a). Questing adults of three tick species of unknown age collected in northeast and southeast Kansas, engaged in water drinking upon arrival to the laboratory: *Amblyomma americanum* [30 out of 56 (24/36 females and 6/20 males)]*, D. variabilis* [20 out of 30 (14/20 females, 6/10 males)], and *A. maculatum* (2/2 females). Ticks that did not exhibit drinking behavior within the first 24–72 hrs after collection, died possibly from desiccation.

### Tick survival with and without access to liquid water

The significance of direct liquid water drinking and water uptake through vapor was clearly shown in *A. americanum* survival. Ticks with free daily access to a water droplet had no mortality during the entire 30 day bioassay period. In contrast, all ticks with no water droplets died within 12 days (Fig. 2b). Ticks with no direct access to the 5 µl water droplet (covered with the screen mesh for blocking the direct access), survived significantly longer than ticks without any liquid water; however, they started dying on day 6 and 100% mortality was reached in 25 days (Fig. 2b). In this treatment, ticks quickly moved to and stayed on top of the mesh covering the water droplet (as close as possible to the droplet) presumably to take advantage of a higher humidity emanating from the droplet. Males and females had a similar pattern of survival; therefore, we combined these data (Fig. 2b). Tick survival was significantly different among the three treatments; water droplets vs. no water (p < 0.0001), water vapor vs. no water (p = 0.0099), and water vapor vs. water droplets (p < 0.0001).

### Quatification of water intake

Using bacteria in a water droplet as quantifiable tracer, we were able to estimate by bacterial cell counts that males (n = 20) ingested 0.20 ± 0.05 µl (*Staphylococcus epidermidis*) and 0.22 ± 0.03 µl (*E. coli*) of water within a period of 60 min. In females (n = 18) water intake was 0.33 ± 0.07 µl (*S. epidermidis*) and 0.32 ± 0.05 µl (*E. coli*) (Table 3). Capillary feeding showed that intake within 60 min was 0.44 ± 0.07 µl (n = 20) and 0.55 ± 0.06 µl (n = 20) in males and females, respectively (Table 3).

**Table 3:** Water intake of *Amblyomma americanum*.

Droplet feeding				Capillary feeding	
*Staphylococcus epidermidis* (4.8 ± 0.7 × 10^3^ CFU/µl)		*Escherichia coli* (7.6 ± 0.7 × 10^3^ CFU/µl)			
**Males (n = 20)**	**Females (n = 18)**	**Males (n = 18)**	**Females (n = 20)**	**Males (n = 20)**	**Females (n = 20)**
0.20 ± 0.05*	0.33 ± 0.07	0.22 ± 0.03	0.32 ± 0.05	0.44 ± 0.07	0.55 ± 0.06

^*^Mean µl ingested ± standard error of mean.

### Internal distribution of ingested water (droplet and capillary feeding)

After 30 min of drinking from a capillary tube, we detected fluorescence in the type I acini of the salivary glands and in the midgut, in both males and females (n = 4 each) (Fig. 1e,g). After drinking from a fluorescent water droplet, all ticks showed fluorescence in type I acini of the salivary glands, whereas only 1 of 2 and 2 of 3 males and females, respectively, showed fluorescence also in the midgut (Fig. 1d,f). This suggests that the uptake of water occurs through both, the salivary glands and the midgut as proposed for *I. scapularis*^17^, whereas the uptake through the salivary glands is the primary mechanism in the voluntary feeding of the water droplet.

### Exploitation of water drinking behavior for tick management

#### Toxicity of ingested ions

The tick osmoregulatory system must have limitations for transport of certain ions. Osmoregulation occurs through ion transport resulting in an electro-osmotic gradient across the cell layers. Therefore, we aimed to identify inorganic compounds that disrupt tick osmoregulation and water balance and lead to high tick mortality within 7 days (Tables 1 and S1).

**Figure 3:**
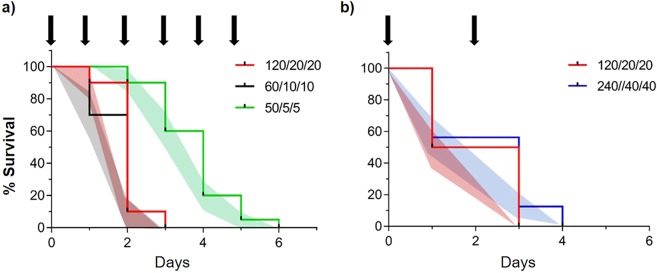
Kaplan-Meier survival curves after lone star ticks water droplet drinking with inorganic compounds. All survival curves show treatments with KH_2_PO_4_ + NaCl + KNO_3_ at different concentrations/ratios and different application frequency. (**a**) Survival of *A. americanum* with concentrations in mM: 120/20/20; 60/10/10; and 50/5/5. Two replicates per treatment male and female ticks (n = 20). (**b**) Survival of *A. americanum* at 2-fold and 4-fold increase in compound concentration at a lower application frequency. Two replicates per treatment of male and female ticks (n = 16). All control ticks (offered water droplet) in each treatment exhibited 100% survival after day 15 (data not shown). Black arrows point to treatment days. Color shaded areas represent SEM for each data point.

Solutions containing potassium phosphate monobasic (KH_2_PO_4_), sodium chloride (NaCl), and potassium nitrate (KNO_3_) resulted in tick mortality in less than 7 days. For the most efficient treatment (60 mM KH_2_PO_4_/10 mM NaCl /10 mM KNO_3_), 100% mortality was achieved by day 3 (Fig. 3a). The lowest concentration to achieve 100% tick mortality in less than 7 days was 50 mM KH_2_PO_4_/5 mM NaCl/5 mM KNO_3_ with a median survival at day 4 and 100% mortality at day 6 (Fig. 3a). A two-fold increase in the ion concentration (120/20/20 mM) did not increase the speed of tick mortality, and the resulting survival curves were not statistically different (p = 0.398) (Fig. 3a). A decrease in treatment application frequency (every other day) using a 2-fold increase in concentration was not significantly different from that of daily applications (p = 0.499 and mortality day 3) (Fig. 3b). Likewise, a 4-fold increase in ion concentration at the same application frequency did not increase mortality (Fig. 3b).

#### Toxicity of ingested microorganisms

Water delivery of various bacteria, fungi, and their toxins was also tested in a search for agents lethal to ticks. Among bacteria, only *Pseudomonas aeruginosa* caused 100% mortality in less than 7 days post-treatment (Table 2). Presence of this bacterium was confirmed in dead ticks after surface sterilization, processing, and plating of individual ticks on trypticase soy agar. Although we observed some tick mortality (day 10 to 15 post-treatment) after treatments with *Bacillus thuringiensis*, entomopathogenic fungi, and *Bt* toxins, high tick mortality was not achieved (Tables 2 and S2).

**Figure 4:**
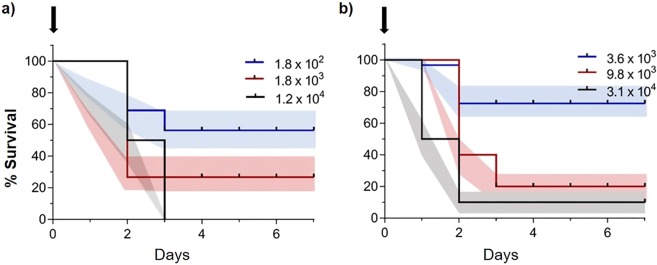
LD50 estimation with Kaplan-Meier survival curves after droplet and capillary drinking of water with *Pseudomonas aeruginosa*. (**a**) Survival of *A. americanum* after droplet feeding. Two replicates per treatment in treatments 1.8 × 10^3^ and 1.8 × 10^2^ (n = 17 and n = 16 respectively). Treatment 1.2 × 10^4^ was conducted only with females, 3 replicates (n = 15); (**b**) Survival of *A. americanum* after capillary feeding. One replicate per treatment, 20 individuals per group (10 males 10 females). Labels represent the inoculum size in CFU per microliter. Black arrows point to the treatment day. Color shaded areas represent the SEM for each data point.

Based on the positive results with *P. aeruginosa*, we aimed to determine the LD_50_ for adult ticks using capillary and droplet drinking. We were able to achieve 100% mortality at day 3 post-treatment with 1.1 × 10^4^ CFU/µl of *P. aeruginosa* in female ticks by droplet drinking with a median survival of 2 days. We were not able to reach 100% mortality with lower bacterial concentrations (Table 2, Fig. 4a). In the capillary feeding approach, the mortality was lower than that in droplet feeding with the maximum mortality 90% in 7 days (Fig. 4b). *Pseudomonas aeruginosa* was recovered from all dead ticks in this bioassay and bacterial concentrations in these ticks was very high (Table S3), while no bacteria were recovered from the control group of ticks.

## Discussion

It has been established for a long time that ticks feed exclusively on host blood as the only source of nutrients. Since ticks spent most of their life off the host, it is critical for them to maintain water balance to prevent desiccation and death^6–9^. Ticks use passive water vapor absorption and active water vapor uptake^6,8,10,16^. In addition, there are several rather dated and sporadic observations that some ticks may actively uptake liquid water^18,20^. For *A. americanum*, Needham and Teel (1986) reported that larvae were seen gathering around and inserting their mouthparts in water droplets. Nonetheless, nothing is known about water ingestion and its significance in tick fitness and survival nor was it explored for potential tick management.

In our study, we describe for the first time adult lone star ticks ingesting liquid water. Active water uptake is shown by our observation of pulsatile movements in the base of hypostome with the mouthparts in the water and the recovery of bacteria and the fluorescent dye mixed in the water from the tick midgut and the salivary glands. More importantly, we show a significant difference in survival between ticks that did and did not have physical contact with water droplets; with no mortality for 30 days (up to 4 months, not shown) and 100% mortality by day 25 (median survival day 9), respectively. As expected, ticks without daily supply of water droplets died even faster with 100% mortality within 12 days. This clearly demonstrates the significance of direct contact with liquid water and water drinking in tick survival. Considering the critical importance of maintaining water balance to prevent desiccation, we propose that the ability of lone star ticks to drink liquid water is likely one of the main factors behind the great success and continuous expansion of this species in the United States^22^. This is supported by modeling studies showing that vapor pressure is one of the most important determinants of tick suitable habitat^23^ and continued expansion of the lone star tick^24^. Nevertheless, additional studies assessing tick survival considering macro-, meso- and microclimatic levels of different habitats are needed to further assess the importance of tick water drinking and its spatial and temporal impacts.

The volume of water ingested differed between males and females and this is likely due to differences in the body size. The water volume measured in droplet feeding using bacteria, as a quantifiable tracer, was lower than that with the capillary feeding and this is likely because of the underestimation of water volume due to digestion of bacteria in the midgut and some loss during bacterial recovery and culturing. These bacteria were recovered from the internal soft tissues and likely derived from the midgut and the salivary glands, as these are the sites for water absorption^17^.

Although other studies reported an increased weight gain of ticks after being in physical contact with water droplets, this was believed to occur mainly due to water vapor absorption through the cuticle^25,26^. Clearly, in our study we demonstrate that water vapor alone is not the main contributing factor for extended survival rates. Previously, Londt and Whitehead^19^ reported in a study from the South Africa that larvae of *A. hebraeum, Rhipicephalus appendiculatus, R. evertsi, R. decoloratus* were observed to imbibe water through the mouthparts from a moist filter paper and these ticks survived longer than those without wet filter paper. However, these authors could not distinguish between water vapor and water drinking. In our previous study on *I. scapularis*^17^, we observed water in the type I acini of the salivary glands and the midgut after capillary feeding. However, water droplet drinking behavior was rare with only 5% (2/43) ticks ingesting water this way. Studies that examined other tick species including *Ixodes ricinus, Dermacentor marginatus*, and *D. reticulatus* reported no liquid water drinking^14–16^. Interestingly, in addition, to *A. americanum* (laboratory-reared and from the field), we observed water drinking behavior in wild *A. maculatum* and *D. variabilis*. However; these results are preliminary and the significance of this behavior in tick biology and fitness remains to be examined.

We also aimed to exploit the water drinking behavior for *A. americanum* management. From the 48 inorganic compound combinations tested, daily applications of water with KH_2_PO_4_ (60 mM) + NaCl (10 mM) + KNO_3_ (10 mM) resulted in 100% mortality of ticks in three days. We also tested two-fold and 4-fold concentration of this solution combined with decrease application frequency (every other day); however, tick mortality was not significantly different from that of the original treatment. We suggest that this is an indication of a threshold of salt concentration at which certain combinations of ions disrupt tick osmoregulation and lead to irreversible dehydration and death. During dry periods, ticks prevent dehydration by reducing water excretion^27^ and by secretion of hyperosmolar saliva rich in Na+, K+ and Cl- capturing water vapor from the air^28^. Interestingly, in our study only assays with phosphates resulted in high tick mortality. It is likely that phosphate salts cause imbalance in ions and water regulation. In vertebrates, inorganic phosphate homeostasis is maintained by two families of phosphate transporters transporting Pi into the cells based on the electrochemical gradient provided by Na+/K+ ATPase^29^. The mechanism for disruption of tick osmoregulation by phosphate salts could be due to a direct effect on the electrochemical gradient or due to disruption of anionic transporters of the excretory system; however, this remains to be further investigated. While this approach needs to be tested in the field, it is conceivable that for example, spraying water with a mix of KH_2_PO_4_, NaCl, and KNO_3_ in small droplets from a regular garden sprayer on a grass lawn, provides an effective control of lone star ticks.

In addition to the inorganic salts, we also tested different microorganisms including bacteria, fungi, and their toxins in search of agents that are lethal to ticks. After only one-time exposure, *P. aeruginosa* caused 100% mortality in ticks within three days post-treatment. Although some tick mortality was observed with other microorganisms tested, none of those led to high tick mortality within 7 days. While *P. aeruginosa* is a general opportunistic pathogen and therefore unsuitable for a biological control of ticks, these data show great potential for using this approach with other bacterial species and strains or their toxins.

## Conclusion

Overall, our results demonstrate that water drinking behavior is common in *A. americanum* and possibly several other ixodid ticks, and has a significant impact on tick survival. In addition, water drinking can be used as a mean to deliver toxic compounds and microorganisms for tick management. We propose the exploitation of the tick water drinking behavior to target important physiological systems, which would make ticks vulnerable to dehydration and microbial dysbiosis.

## Supplementary information

Supplementary Information.
