# Supplementary material for: Liquid water intake of the lone star tick, Amblyomma americanum: Implications for tick survival and management
Source: Sci Rep. 2020 Apr 7;10:6000. doi: 10.1038/s41598-020-63004-9 (PMC7138852; doi:10.1038/s41598-020-63004-9)
Supplement: Supplementary file 1 — Supplementary Information. [file 41598_2020_63004_MOESM1_ESM.pdf]

Supplementary data

Liquid water intake of the lone star tick, *Amblyomma americanum*: Implications for tick survival and management

L. Paulina Maldonado-Ruiz[1], Yoonseong Park[1], and Ludek Zurek [2,3*]

Supplementary data

Table S1. Inorganic compounds tested and their impact on *Amblyomma americanum* survival after water droplet ingestion

			Males		Females		Combined	
**Treatment**	**Final concentration mM (compound ratio)**	**Length of bioassay (days)**	***n***	**% survival (days)**	***n***	**% survival (days)**	***n***	**% survival (days)**
**NaCl**	100	10	10	100 (10)	10	100	20	100
**KH_2PO_4 + NaCl**	500 (1:1)	7	8	0 (3)	8	0 (5)	16	0 (5)
**KH_2PO_4 + NaCl**	250 (1:1)	7	5	0 (4)	5	0 (3)	10	0 (4)
**KH_2PO_4 + NaCl**	125 (1:1)	7	5	0 (6)	5	0 (4)	10	0 (6)
**KH_2PO_4 + NaCl**	25 (1:1)	7	5	100 (7)	5	100	10	100
**KH_2PO_4 + NaCl**	20 (1:1)	7	5	60 (7)	5	80	10	70
**KH_2PO_4 + NaCl**	125/62.5	7	5	0 (3)	5	0 (4)	10	0 (4)
**KH_2PO_4 + NaCl**	125/30.12	7	5	0 (6)	5	0 (4)	10	0 (6)
**KH_2PO_4 + NaCl**	125/15	7	5	0 (4)	5	0 (3)	10	0 (4)
**KH_2PO_4 + NaCl**	62.5/15	7	5	0 (6)	5	0 (7)	10	0 (7)
**ATP**	10	19	10	50 (19)	10	90	20	70
**Dopamine**	10	19	10	80 (19)	10	50	20	65
**Caffeine**	10	19	9	0 (17)	10	20	19	10
**ATP + NaCl**	10/500	19	10	0 (7)	10	0 (9)	20	0 (9)
**Dopamine + NaCl**	10/500	19	10	0 (6)	10	0 (6)	20	0 (6)
**Caffeine + NaCl**	10/500	19	10	0 (4)	10	0 (7)	20	0 (7)
**KH_2PO_4 + NaCl**	60/10	7	5	0 (3)	5	20	10	10
**KH_2PO_4 + NaCl**	60/5	7	5	20 (7)	5	0 (4)	10	10
**KH_2PO_4 + NaCl + KNO_3**	60/10/5	7	5	20 (7)	5	0 (2)	10	10
**NH_4N0_3 + NaCl**	50/5	7	10	100 (7)	10	100	20	100
**[1]KH_2PO_4 + NaCl + KNO_3**	60/5/10	7	5	0 (5)	5	20	10	10
**[1]KH_2PO_4 + NaCl + KNO_3**	30/5/10	7	5	80 (7)	5	20	10	50
**[1]KH_2PO_4 + NaCl + KNO_3**	30/5/5	7	5	40 (7)	5	80	10	60
**[1]NH_4N0_3**	20	7	5	100 (7)	5	80	10	90
**[1]$(NH_4)_2PO_4$**	20	7	5	100 (7)	5	80	10	90
**[1]NH_4N0_3 + NaCl**	20/10	7	5	40 (7)	5	100	10	70

[ł]**NH_4N0_3 + NaCl**	20/5	7	5	60 (7)	5	100	10	80
[ł]**$(NH_4)_2PO_4$ +NaCl**	30/5	7	5	40 (7)	5	60	10	50
[ł]**$(NH_4)_2PO_4$ +NaCl**	20/10	7	5	60 (7)	5	40	10	60
[ł]**$(NH_4)_2PO_4$ +NaCl**	20/5	7	5	80 (7)	5	80	10	80

[ł] Every other day treatments

Table S2. Microorganisms and their impact on *Amblyomma americanum* survival after water droplet ingestion

**Treatment**	**Concentration (CFU/µl)**	**Length of bioassay**	***n* (♂,♀)**	**% survival**
		**Microorganisms**		
Bacillus thuringiensis				
**Cry 4B**	7.6 x 10^4	10	10 (5,5)	100
**Cry 11A**	3.7 x 10^4	10	10 (5,5)	100
Beauveria bassiana				
**high concentration**	unknown	10	20 (10,10)	100
**low concentration**	unknown	10	9 (9,0)	88.9
***high concentration**	unknown	7	10 (10,0)	70
***low concentration**	unknown	7	10 (10,0)	90
Isaria sinclairii				
**high concentration**	unknown	7	5 (5,0)	100
		**Toxins**		
**Protein toxin A**	0.2 mg/ml	10	16 (8,8)	100
**Protein toxin B**	0.6 mg/ml	10	16 (8,8)	100

*bioassays with nymphs

Table S3. Mortality of *A. americanum* after ingestion of *Pseudomonas aeruginosa* through capillary feeding

Inoculum CFU/µL	Ingested cells ± SEM	+Mortality (%)	*P. aeruginosa* confirmation (%)*
$3.1x10^4$	$1.5 \pm 0.2 \times 10^4$	90	100
$9.8x10^3$	$3.2 \pm 0.4 \times 10^3$	80	100
$3.6x10^3$	$1.3 \pm 0.2 \times 10^3$	30	100

+Mortality (%) for each inoculum was calculated from 20 individuals tested (10 males and 10 females)
*Percentage calculated from the total dead individuals.
